# A high-quality, chromosome-level genome assembly of the Black Soldier Fly (*Hermetia illucens* L.)

**DOI:** 10.1093/g3journal/jkab085

**Published:** 2021-04-12

**Authors:** Tomas N Generalovic, Shane A McCarthy, Ian A Warren, Jonathan M D Wood, James Torrance, Ying Sims, Michael Quail, Kerstin Howe, Miha Pipan, Richard Durbin, Chris D Jiggins

**Affiliations:** 1 Department of Zoology, University of Cambridge, Cambridge CB2 3EJ, UK; 2 Wellcome Sanger Institute, Wellcome Trust Genome Campus, Hinxton, Cambridge CB10 1SA, UK; 3 Department of Genetics, University of Cambridge, Cambridge CB2 3EH, UK; 4 Better Origin, Entomics Biosystems Limited, Cambridge CB3 0ES, UK

**Keywords:** *Hermetia illucens*, Black Soldier Fly, genome assembly, Hi-C assembly, PacBio, BRAKER2, genome annotation, inbreeding, domestication

## Abstract

*Hermetia illucens* L. (Diptera: Stratiomyidae), the Black Soldier Fly (BSF) is an increasingly important species for bioconversion of organic material into animal feed. We generated a high-quality chromosome-scale genome assembly of the BSF using Pacific Bioscience, 10X Genomics linked read and high-throughput chromosome conformation capture sequencing technology. Scaffolding the final assembly with Hi-C data produced a highly contiguous 1.01 Gb genome with 99.75% of scaffolds assembled into pseudochromosomes representing seven chromosomes with 16.01 Mb contig and 180.46 Mb scaffold N50 values. The highly complete genome obtained a Benchmarking Universal Single-Copy Orthologs (BUSCO) completeness of 98.6%. We masked 67.32% of the genome as repetitive sequences and annotated a total of 16,478 protein-coding genes using the BRAKER2 pipeline. We analyzed an established lab population to investigate the genomic variation and architecture of the BSF revealing six autosomes and an X chromosome. Additionally, we estimated the inbreeding coefficient (1.9%) of the lab population by assessing runs of homozygosity. This provided evidence for inbreeding events including long runs of homozygosity on chromosome 5. The release of this novel chromosome-scale BSF genome assembly will provide an improved resource for further genomic studies, functional characterization of genes of interest and genetic modification of this economically important species.

## Introduction

**Figure 1:**
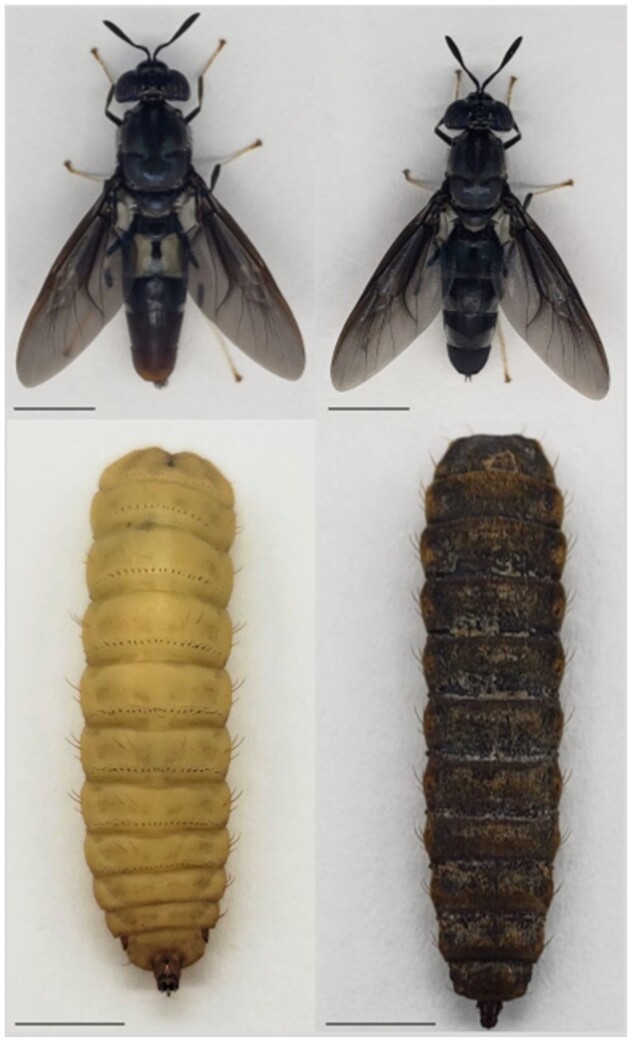
*Hermetia illucens* key life stages. Dorsal view of *Hermetia illucens* adult male (upper left), adult female (upper right), larvae (5th instar; lower left), and pupa (lower right). Adult sex identified by genital shape and posterior color. Scale bar = 5 mm. Photos: T.N. Generalovic.

The Black Soldier Fly (BSF; Figure 1), *Hermetia illucens*, Linnaeus, 1758 (Diptera: Stratiomyidae; NCBI: txid343691) is a species of growing interest in both bioremediation and the production of food and feed. Endemic to tropical and sub-tropical regions of the Americas, BSF is now distributed globally extending to temperate regions of Europe and Asia through commercialization and human-mediated expansion (May 1961; Sheppard *et al.* 2002; Roháček and Hora 2013). The increasing popularity of BSF in insect farming is due to the high feed-to-protein bioconversion rates of BSF larvae coupled with a generalist diet. The conversion efficiency of BSF larva is higher than other traditional edible insects such as *Tenebrio molitor* (Yellow mealworm) (Oonincx *et al.* 2015). Due to the high protein (40%) and lipid (35%) content of BSF larvae, they are now a European Union approved feed ingredient in aquaculture and poultry farms as a replacement to inefficient and unsustainable fish and soybean meal (St-Hilaire *et al.* 2007; Kroeckel *et al.* 2012). Additionally, the rich biomass of BSF larvae has led to the resource exploitation of lipids and chitin for the cosmetic industry, as a source of biofuel production and recently shown promise as a source of antimicrobials (Li *et al.* 2011; Park *et al.* 2015; Rabani *et al.* 2019; Purkayastha and Sarkar 2020). With short generation times, large brood sizes (∼900 eggs per clutch), and voracious feeding behavior, the BSF is the optimal species for insect mass rearing (Booth and Sheppard 1984; Shishkov *et al.* 2019).

Global food security and waste management systems are increasingly under threat from a growing human population. An increase in food production to feed a population of over 9 billion by 2050 will require a demand for protein over 270 million tons (Gustavsson *et al.* 2011; Food and Agriculture Organization of the United Nations 2013). With one-third of this produce likely to be processed as food waste, a transition to a more sustainable agricultural model is essential (Gustavsson *et al.* 2011). Shifting to a circular bioeconomy utilizing insect biomass can lead to a more sustainable global food industry (Barragan-Fonseca *et al.* 2017). With high-nutrient content and the ability to upcycle organic waste streams, the BSF is the most exploited species in the growing insect farming industry yet there remains a lack of research on the genetics of the BSF (Tomberlin and van Huis 2020).

While genomic resources within the Diptera are well established through databases such as FlyBase, the resources available for BSF are limited (Thurmond *et al.* 2019). A reference genome of the BSF has recently been released but is relatively fragmented (Zhan *et al.* 2020). The expanding industry of BSF farming must rapidly match the high-genomic standards of other agricultural systems if it is to become a well-established practice (Warr *et al.* 2020). Recent advances in sequencing technology and high-throughput projects including the Darwin Tree of Life (https://www.sanger.ac.uk/science/collaboration/darwin-tree-life-project) have enabled the assembly of many non-model reference genomes (Amarasinghe *et al.* 2020). Arthropod genome assembly projects can be hindered by limited starting material, ploidy level, repeat-rich genomes, and high heterozygosity (Dominguez Del Angel *et al.* 2018). Resolving genome heterozygosity remains a major challenge for genome assembly projects (Michael and VanBuren 2020). Nonetheless, integrating long read and linked-read sequencing has greatly facilitated *de novo* assemblies (Elyanow *et al.* 2018). Combined with high-throughput chromosome conformation capture (Hi-C) sequencing, the assembly of chromosome-level reference genomes in vertebrates, invertebrates, and plants is far more achievable (Lu *et al.* 2019; Wang *et al.* 2019; Ning *et al.* 2020).

Here, we present a chromosome-scale, 1.01 Gb genome assembly for *H. illucens* (BSF). We use a combination of Pacific Bioscience (PacBio) long read, 10X Genomics Linked read and chromosomal conformation capture sequence data to assemble a highly contiguous and complete genome. We use genome re-sequence data to identify a sex chromosome, assess the genomic variation, and the level of inbreeding in an established lab population. As the first chromosome-scale assembly available for the BSF this resource will enable the genetic characterization of performance traits and will further the development of genetic studies.

## Materials and methods

### Insect husbandry and collection

A captive population of *Hermetia illucens* was supplied by Better Origin (Entomics Biosystems Limited, UK) from a previously farmed European (UK) population provided by commercial suppliers which were subsequently bolstered with an Asian lineage. This population was maintained for multiple generations at the University of Cambridge, Zoology department (Cambridge, UK). A mating pair was isolated, and the offspring reared under conditions of 29 ± 0.8°C, 60% relative humidity on a 16:8-hour light: dark cycle. Larval offspring were fed twice weekly *ad libitum* on Better Origin (Cambridge, UK) “BSF Opti-Feed” mixed 30:70 with non-sterile H_2_O under the same conditions. Pre-pupae were transferred to medium-grade vermiculite (Sinclair Pro, UK) for pupation and emerged adults moved to a breeding cage (47.5×47.5×93 cm; 160 µm aperture). An adult female and male of the founding population and two offspring pupae were collected and stored at −80°C until processed for sequencing. Additional offspring from the same pair were used to establish a BSF colony line named “EVE” for future analysis. A sample (*n* = 12) of the EVE colony from the eighth-generation post-lab establishment was also collected and stored at −80°C until processed for sequencing.

### Genome size and heterozygosity estimates

Genomic DNA (gDNA) was extracted from the thorax of the mature adults using the Blood & Cell Culture DNA Midi Kit (Qiagen, Germany) following the manufacturers’ protocol. Paired-End (PE) libraries were produced and sequenced on the Illumina HiSeq X Ten platform (Illumina, United States) at the Wellcome Sanger Institute (Cambridge, UK). Sequencing data were used to estimate genome size, heterozygosity, and genomic repeats using GenomeScope (Vurture *et al.* 2017).

### Genome library construction and sequencing

An offspring pupa from the isolated pair was collected to prepare libraries for both Pacific Biosciences (United States) and 10X Genomics Chromium linked-read (10X Genomics, United states) sequencing (Supplementary Table S1). All *de novo* genome sequencing was carried out at the Wellcome Sanger Institute (Cambridge, UK). High-molecular-weight DNA was extracted from the pupal offspring using a modified MagAttract HMW DNA protocol (Qiagen, Germany) and a PacBio Single Molecule Real-Time (SMRT) sequencing library was prepared. Eight SMRT cells (1 M v2) were sequenced using version 2.1 chemistry of the PacBio Sequel platform. An additional sequencing library was constructed and sequenced on a single 8 M SMRT cell on the Sequel II platform using version 0.9 sequencing chemistry. A 10X Genomics Chromium linked read 150 bp PE library was also prepared from the gDNA of the same individual and sequenced on the Illumina HiSeq X Ten platform (Illumina, United States). A Hi-C PE library was constructed from the tissue of a sibling pupa and 150 bp PE reads were sequenced on the Illumina HiSeq X Ten platform.

### Genome assembly

**Figure 2:**
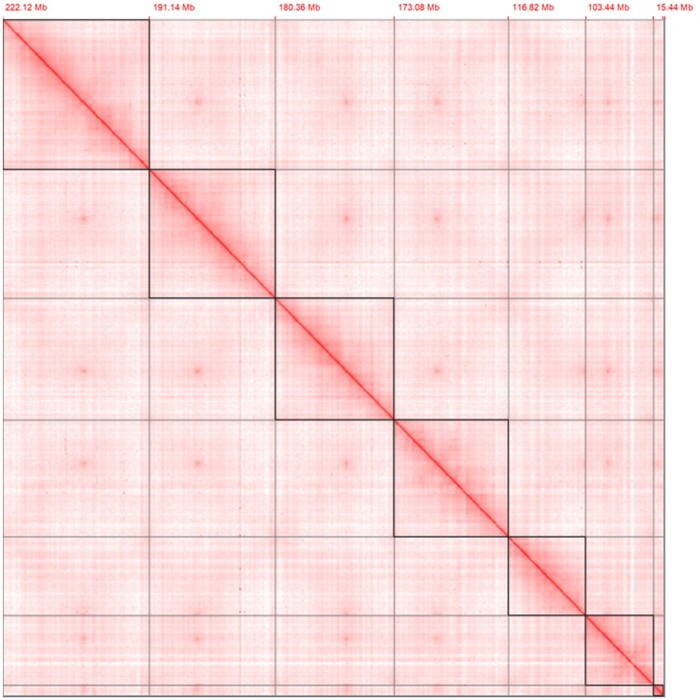
Curated Hi-C contact map of chromosomal interactions. Super-scaffold chromosomes (*n* = 7) are highlighted within black frames and annotated with assembled length at the beginning of each chromosome (interactive map available on https://higlass-grit.sanger.ac.uk/l/?d=OjQNRJcmTgKyKfvkk3yODg).

Due to the high-predicted heterozygosity of the BSF genome, we employed FALCON-Unzip, a *de novo* diploid-aware assembler of PacBio SMRT sequence data before scaffolding (Chin *et al.* 2016). The FALCON-Unzip algorithm utilizes the hierarchical genome assembly process (HGAP) enabling haplotype resolution. Raw PacBio data containing reads longer than 5 kb were selected for error correction and consensus calling. The FALCON-Unzip draft assembly was purged of duplicate sequences using purge_dups v0.0.3 (Guan *et al.* 2020). The 10X linked reads were used to scaffold the draft FALCON-Unzip assembly using scaff10x (https://github.com/wtsi-hpag/Scaff10X). The assembly was polished with one round of arrow (https://github.com/PacificBiosciences/GenomicConsensus) using the error corrected reads of FALCON-Unzip. The Hi-C data were mapped to the intermediate assembly in the second round of scaffolding using BWA (Li and Durbin 2009). A Hi-C contact map was generated and visualized in HiGlass (Kerpedjiev *et al.* 2018). The final draft assembly was manually curated to remove contaminants, correct structural integrity, assemble and identify chromosome-level scaffolds (Figure 2) based on gEVAL analyses and 3 D-chromosomal interactions (Howe *et al.* 2021). The remaining haplotype duplication was purged manually into an alternative haplotype genome.

### Genome quality evaluation

To assess the quality of the reported assembly, we evaluated both genome completeness and contamination. We used BUSCO v3.0.2 (Benchmarking Universal Single-Copy Orthologs) (Simão *et al.* 2015) to identify conserved genes within the “insecta_odb9” and “diptera_odb9” databases. Contamination was assessed using the BlobToolKit environment v1.0 (Challis *et al.* 2020) to screen for contaminant sequence.

### Repeat sequence identification and genome annotation

To quantify the repetitive regions within the BSF genome, we modeled and masked a *de novo* library of repetitive sequences using RepeatModeler v2.0.1 and RepeatMasker v4.0.9 (Smit *et al.* 2015). A custom repeat consensus database was built, and repeat elements classified using RepeatModeler (-engine NCBI). The Dfam_Consensus-20181026 (Hubley *et al.* 2016) and RepBase-20181026 (Bao *et al.* 2015) databases were combined with the custom repeat database. Using the merged database, RepeatMasker was used to identify and soft mask repetitive regions using the RMBlast v2.6.0 sequence search engine.

Genome annotation of the assembly was produced using the BRAKER2 pipeline v2.1.5 (Altschul *et al.* 1990; Stanke *et al.* 2006, 2008; Camacho *et al.* 2009; Barnett *et al.* 2011; Hoff *et al.* 2016, 2019; Brůna *et al.* 2020a, 2020b). Published RNA-seq reads were used for annotation, obtained from whole larva (study accession: PRJEB19091) (Vogel *et al.* 2018) and a separate study using the full BSF life cycle; egg (12 & 72 hours-old), larva (4, 8 & 12 days-old), pre-pupa, and pupa (both early and late stages) including both male and female adults (BioProject ID: PRJNA573413) (Zhan *et al.* 2020). Arthropod proteins were obtained from OrthoDB (Kriventseva *et al.* 2019). RNA-seq reads were filtered for adapter contamination and low-quality reads using Trimmomatic v0.39 (Bolger *et al.* 2014) followed by quality control pre and post trimming with fastqc v0.11.8 (Andrews 2010). RNA-seq reads were mapped to the assembly using STAR (Spliced Transcripts Alignment to a Reference) v2.7.1 (Dobin *et al.* 2013) in 2-pass mode. Protein hints were prepared as part of the BRAKER2 pipeline using ProtHint v2.5.0 (Brůna *et al.* 2020b). BRAKER2 *ab initio* gene predictions were carried out using homologous protein and *de novo* RNA-seq evidence using Augustus v3.3.3 (Hoff *et al.* 2016) and GeneMark-ET v4.38 (Stanke *et al.* 2006). Genome annotations were assessed for completeness using BUSCO v3.0.2 (–m prot) “insecta_odb9” and “diptera_odb9” databases (Simão *et al.* 2015).

### Comparative genome assembly analysis

We evaluated the assembly statistics of the iHerIll genome for completeness, contiguity, and correctness with related Diptera species and the only published BSF reference, GCA_009835165.1 (Zhan *et al.* 2020). Assembly statistics for publicly available Diptera genomes were generated using assembly-stats (https://github.com/sanger-pathogens/assembly-stats). To assess the completeness of the iHerIll assembly, we used BUSCO v3.0.2 “insecta_odb9” and “diptera_odb9” core gene sets and compared results with related Diptera.

To measure genome contiguity and correctness, we incorporated a quality assessment tool, QUAST v5.1.0 (Gurevich *et al.* 2013). The GCA_009835165.1 assembly was first filtered to purge highly fragmented contigs <10 kb, retaining 99.86% of the original sequence and aligned to the reported iHerIll assembly. Genome alignment statistics generated using QUAST (–large –eukaryote –min-alignment 500 –extensive-mis-size 7000 –min-identity 95 –k-mer-stats –k-mer-size 31 –fragmented) provided NA50 and LA50 metrics based on aligned sequences, enabling comparable results to be drawn between the two assemblies and identify potential misassembly events. We next produced whole genome alignments using mummer v3.23 (Kurtz *et al.* 2004) and visualized alignments using dnanexus (https://dnanexus.github.io/dot/). To test whether the BSF reference assemblies harbored a unique genomic sequence, we hard masked both the genomes using the custom repeat library for re-alignment.

### Genomic variation

A sample of 12 individuals from generation eight of the sampled BSF line “EVE” was sequenced on the BGI-seq platform. DNA from adult whole thorax tissue was extracted using the Blood & Cell Culture DNA Midi Kit (Qiagen, Germany). Sequencing libraries for 12 individuals of 150 bp PE PCR-free BGI-seq Whole Genome Sequencing (WGS) were prepared and sequenced to an average genome coverage of 25× by BGI (BGI, Hong Kong). Sequencing data were quality checked using FastQC v0.11.5 (Andrews 2010) pre and post adapter trimming with cutadapt v1.8.1 (Martin 2011). Raw data were mapped to the assembled genome using BWA v0.7.17 (Li and Durbin 2009), sorted with samtools v1.9 (Li *et al.* 2009), and duplicates removed using picard v2.9.2 (http://broadinstitute.github.io/picard). Variant calling was carried out using bcftools mpileup v1.9 and filtered using vcftools v0.1.15 (Danecek *et al.* 2011; Li 2011). To obtain high-quality Single Nucleotide Polymorphism (SNP) datasets, we removed indels (–remove-indels), applied a minimum and maximum mean depth (–min-mean-DP 12; –minDP 12; –max-meanDP 30; –maxDP 30), a minimum quality threshold (–minQ 30), and removed sites missing >15% of data (–max-missing 0.85). Genome nucleotide diversity (π) and Tajima D were calculated over 20 kb windows using popgenWindows.py (-w 20000 -m 100 -s 20000) (https://github.com/simonhmartin/genomics_general) and vcftools (–TajimaD 20000), respectively. Further filtering for minor allele frequency (MAF) filter (–maf 0.05) was also applied for runs of homozygosity (ROH) analysis. Runs of homozygosity (ROH) were generated with the detectRUNS v0.98 R package (Biscarini *et al.* 2019) using sliding windows (windowSize = 15; threshold = 0.05; minSNP = 15, minLengthBps = 200000) and default parameters. Inbreeding coefficients were generated using the calculation $F_{\mathrm{ROH}}=\;\frac{\sum L_{\mathrm{ROH}}}{L}$, where *L* is total autosome length.

### Functional annotation of genomic regions of interest

Additional functional annotation was carried out for a 17.6 Mb region on chromosome 5 putatively under selection. Annotation was performed using the Blast2GO tool (tblastx *e*-value 1.0e-^10^) and the NCBI database (nr) filtered for the taxonomic group Diptera with default parameters (Gotz *et al.* 2008). The same region was also functionally annotated for one-to-one orthologs of the EggNOG database v5.0.0 using EggNOG-Mapper v1.0.3 (Huerta-Cepas *et al.* 2019).

### Sex chromosome identification

We next identified the sex chromosome using re-sequence data from both female (*n* = 7) and male (*n* = 5) adults. The final assembly was hard masked of repetitive sequence using the custom repeat library. Male and female sequence data were mapped using BWA v0.7.17 (Li and Durbin 2009) and merged using samtools v1.9 (Li *et al.* 2009). Mean depth of coverage was generated over 20 kb genome-wide windows in 20 kb steps using samtools v1.9 (Li *et al.* 2009) (depth -aa) including unmapped regions, genomics.py and windowscan.py (–writeFailedWindows -w 20000 -s 20000) (https://github.com/simonhmartin/genomics_general). Mean depth was plotted as log2-fold change (male/female) and visualized in R v3.3.2 (RStudio Team 2019) using the ggplot2 package (Wickham 2009). Genes on the sex chromosome were also functionally annotated as described above.

### Data availability

Raw data sets supporting the results of this article are available under the Bioprojects PRJEB23696 and PRJEB37575. The genome reference generated in this study was submitted to ENA under GenBank: GCA_905115235.1. Publicly available RNA sequencing data were obtained from accession numbers PRJEB19091 and PRJNA573413. Supplementary material is available at figshare: https://doi.org/10.25387/g3.14069669.

## Results and discussion

### Genome size and heterozygosity estimates

Sequencing of the adult female and male produced 150.38 Gb and 162.42 Gb, respectively (Supplementary Table S1). Distribution of k-mers in both individuals using *k *= 31 produced a diploid peak set. We estimated genome size of 1.06 Gb, provisional repeat content of 46.25%, and mean heterozygosity of 1.81% (Supplementary Figure S1 and Supplementary Table S2).

### Genome library construction and sequencing

PacBio sequencing of eight SMRT cells (1Mv2) generated 75.17 Gb subreads with an N50 of 22.71 kb. Sequencing of an additional SMRT cell (8 M) produced 105.69 Gb of data with an N50 of 14.58 kb from the same individual, giving a total of 180.86 Gb. Linked-read 10X and Hi-C sequencing on the HiSeq X Ten platform produced 149.78 Gb and 144.5 Gb, respectively, of raw data.

### Genome assembly

Initial assembly using the FALCON-Unzip algorithm on the raw PacBio data assembled a draft genome size of 1.09 Gb into 140 contigs with an N50 value of 13.7 Mb. Following duplicate purging, polishing, chromosome-scaffolding and manual curation, the resulting chromosome-level assembly named “iHerIll” consisted of a total length of 1.01 Gb, contig N50 of 16.01 Mb, and a scaffold N50 of 180.36 Mb (Table 1; Supplementary Table S3). We anchored 99.75% of assembled scaffolds into seven chromosomes leaving 13 unplaced scaffolds (2.53 Mb; 0.25%; Supplementary Table S4).

**Table 1:** Summary statistics of *Hermetia illucens* and selection of Diptera genomes

Species name	Genome size	Scaffold number	Contig N50	Scaffold N50	Gaps	N count	GC (%)	BUSCO (%)				
								C	S	D	F	M
*Hermetia illucens* (iHerIll)	1.01 Gb	20	16.01 Mb	180.36 Mb	112	26,439	42.47	98.60	97.80	0.80	0.50	0.90
*Hermetia illucens* (GCA_009835165.1)	1.10 Gb	2,806	231.07 kb	1.70 Mb	24,796	14,305,322	42.46	98.90	91.10	7.80	0.60	0.50
*Drosophila melanogaster* (GCA_000001215.4)	143.73 Mb	1,870	21.49 Mb	25.29 Mb	572	1,152,978	41.67	99.70	99.00	0.70	0.20	0.10
*Drosophila virilis* (GCA_000005245.1)	169.7 Mb	13,530	5.10 Mb	31.08 Mb	166	16,600	40.43	99.10	98.10	1.00	0.40	0.50
*Musca domestica* (GCA_000371365.1)	750.40 Mb	20,487	11.81 kb	226.57 kb	102,610	58,683,792	32.37	98.60	96.90	1.70	0.40	1.00
*Stomoxys calcitrans* (GCA_001015335.1)	971.19 Mb	12,042	11.31 kb	504.65 kb	129,113	150,529,258	40.30	98.40	97.70	0.70	1.00	0.60
*Glossina morsitans morsitans* (GCA_001077435.1)	363.11 Mb	24,071^a^	49.77 kb	NA	0	0	34.12	98.90	96.60	2.30	0.60	0.50
*Aedes aegypti* (GCA_002204515.1)	1.28 Gb	2,310	11.76 Mb	409.78 kb	229	22,935	38.18	98.90	94.50	4.40	0.40	0.70
*Culex quinquefasciatus* (GCA_000209185.1)	579.04 Mb	3,171	28.55 kb	486.76 kb	45,500	39,082,744	34.89	96.70	91.80	4.90	0.80	2.50

Assembly statistics for Diptera genomes generated using assembly stats script on the associated reference genome. BUSCO score generated from the “insecta_odb9” database (*n* = 1,658). BUSCO statistics C, complete; S, single-copy; D, duplicated; F, fragmented; M, missing.

a Denotes contig number where the assembly contains no scaffolds.

### Genome quality evaluation

**Figure 3:**
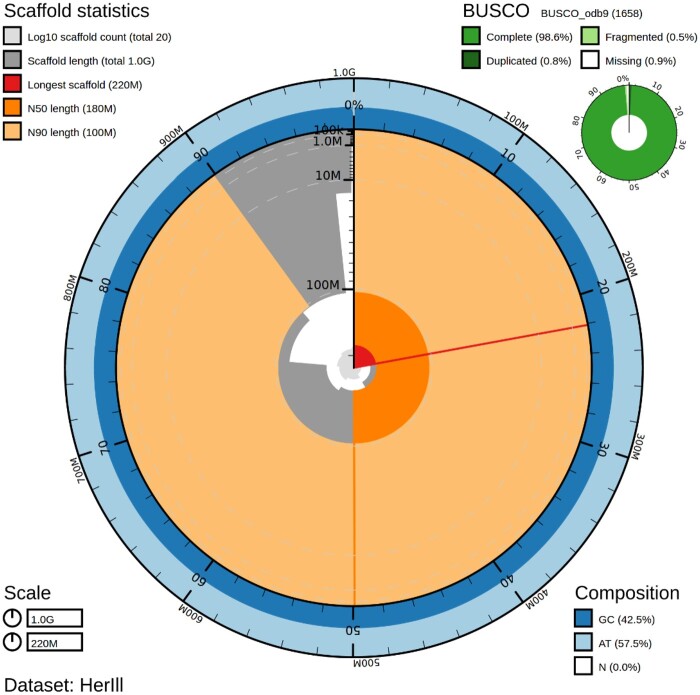
BlobToolKit snail plot of the *Hermetia illucens* assembly. Genome assembly statistics of iHerIll visualized as a snail plot containing BUSCO “insecta_odb9” completeness scores, scaffold assembly statistics, and sequence composition proportions.

The final assembly covered 98.60% and 92.60% of the Insecta and Diptera BUSCO core genes, respectively (Table 1). Within the Insecta BUSCO, completeness score of the reported genome 1622 (97.80%) were single copy with just 15 (0.9%) missing (Figure 3; Supplementary Table S5). BlobToolKit identified 99.89% of the raw PacBio data as exclusively arthropod sequence (Supplementary Figure S2). Arthropoda was the most abundant identified phyla, with a further 1,137,222 bp (0.11%) showing no significant taxonomic identification. Therefore, our assembly does not include any significant assembled contaminate sequences. Whereas the majority of the genome sequence was identified as Diptera (77.83%), segments of closest sequence similarity to Siphonaptera (11.63%), Hymenoptera (10.29%), Coleoptera (0.09%), and Lepidoptera (0.05%) were also identified.

### Repeat sequence identification and genome annotation

Repeat masking resulted in a total of 67.32% (676,593,256 bp) of the genome being identified as repeat regions (Table 2). We identified Long Interspersed Nuclear Elements (LINEs; 44.85%) as the most abundant class of repetitive elements followed by a high proportion of unclassified repeats (13.81%). This repeat analysis identifies comparable statistics to the identified repeats of the previously published BSF genome (65.76%). The BRAKER2 pipeline annotated 16,478 protein-coding genes and 17,664 transcripts which provided BUSCO completeness scores of 98.2% and 95.6% for Insecta and Diptera core gene datasets, respectively (Table 2).

**Table 2:** Genome annotation and repeat statistics of the *Hermetia illucens* assembly

Genome annotation statistics		*Hermetia illucens* assembly	#
Protein-coding genes			16,478
Transcripts			17,664


BUSCO statistics for protein-coding gene annotation		%	#
Insecta (*n* : 1658)	Completeness	98.20	1,627
	*Complete Single-copy*	*91.10*	*1,510*
	*Complete Duplicated*	*7.10*	*117*
	Fragmented	0.70	12
	Missing	1.10	19
Diptera (*n* : 2799)	Completeness	95.60	2,674
	*Complete Single-copy*	*86.10*	*2,409*
	*Complete Duplicated*	*9.50*	*265*
	Fragmented	2.50	70
	Missing	1.90	55

Repeat statistics	bp	%	#
DNA elements	38,102,124	3.79	108,133
LTR	45,335,874	4.51	72,542
LINES	450,717,046	44.85	946,086
SINES	3,620,717	0.36	16,138
Unclassified	138,817,495	13.81	485,192
Total interspersed repeats	676,593,256	67.32	1,628,091

Annotation statistics for *H. illucens* assembly. BUSCO scores generated from the “insecta_odb9” and “diptera_odb9” databases .

### Comparative genome assembly analysis

The BUSCO results were comparable between existing high-quality Diptera genomes and iHerIll (Table 1). In comparison with the previous BSF GCA_009835165.1 assembly, there were 15 BUSCO missing from the iHerIll assembly, whereas seven were missing in the GCA_009835165.1 assembly (Supplementary Table S5). We assembled a high proportion of single-copy orthologs (97.8%) with little gene duplication (0.8%). Many more duplicated genes were seen in the GCA_009835165.1 assembly (7.8%), likely due to unresolved heterozygous regions. Our iHerIll assembly is one of the highest quality assembled dipteran genomes available, assembled into the smallest number of scaffolds with the largest scaffold N50 value amongst sampled Diptera (Table 1). We confirm the much higher contiguity of iHerIll compared with GCA_009835165.1 (Supplementary Figure S3) and identify 787 potentially misassembled contigs in GCA_009835165.1, together with 10 contigs that do not align at all to iHerIll (Supplementary Table S6). The GCA_009835165.1 assembly showed substantial full-length alignment to iHerIll scaffolds but with several insertions and deletions between the assemblies (Supplementary Figure S4). The GCA_009835165.1 assembly contained 302.4 kb of non-repetitive DNA sequence that did not align to the iHerIll assembly. None of the iHerIll contigs failed to align to GCA_009835165.1. Severe inbreeding effects, hybridization, and extensive chromosomal rearrangements may promote high levels of sequence divergence between populations (Baack and Rieseberg 2007). It is therefore likely that some of the contigs that fail to align properly represent true biological differences rather than misassemblies. A previous study of the BSF mitochondrial cytochrome c oxidase I (*CO1*) gene indicated a high level of genetic diversity that is suggestive of a diverse species complex (Ståhls *et al.* 2020). Assembly of further high-quality genomes from additional BSF lines will be essential to reveal the extent of genomic diversity within the BSF species-complex.

### Genomic variation

**Figure 4:**
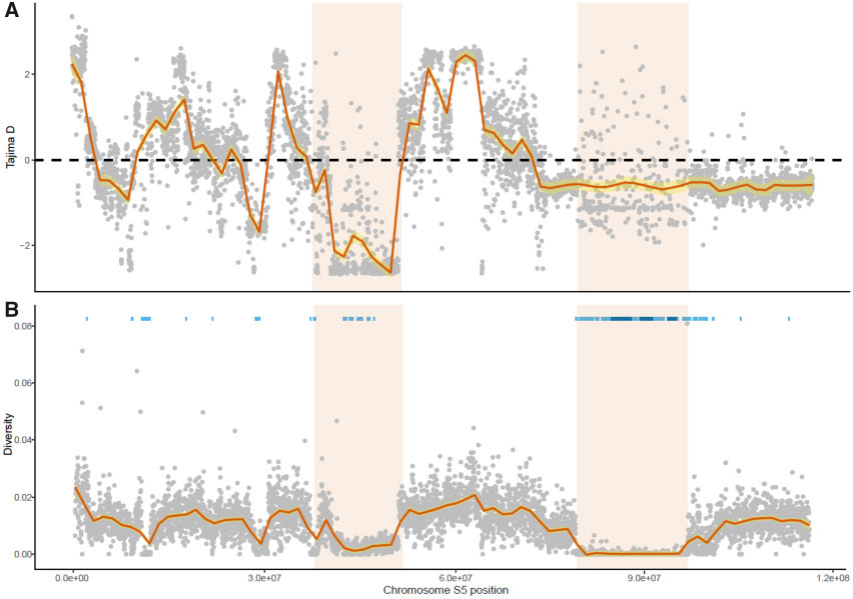
Genome-wide diversity, Tajima D and ROH analysis on chromosome five of the *Hermetia illucens* EVE population. Tajima D (A), nucleotide diversity (B), and Runs of Homozygosity (ROH) (B) values across genomic positions of chromosome five. Mean 20 kb windows smoothed with local regression and regions of interest highlighted. Both short (light blue) and long (dark blue) ROH is shown across genome position.

We estimated the mean genome-wide nucleotide diversity (*π*) as 0.017 and Tajima’s D to be 1.58 (Supplementary Table S7). Chromosome five exhibited the only negative Tajima’s D and the lowest nucleotide diversity. We identified a total of 444 genome-wide ROH in the sample population using 3,834,541 high-quality SNPs of which 96.4% were < 1 Mbp in length (Supplementary Table S7). The remaining 3.6% were deemed long ROH at a length ≥1 Mbp. Islands of ROH were consistent across individuals within the population. Mean ROH length in the EVE line was identified as 393,812 bp with 43.5% of the total runs located on chromosomefive (Supplementary Table S7; Supplementary Figure S5). Half of the identified long ROH were located within a 17.6 Mb region on chromosome five containing 239 annotated genes. The genome-wide inbreeding coefficient estimated from these ROH was 0.019 (1.9%; excluding the identified sex chromosome, see below). This established captive EVE line does not appear to be hindered by inbreeding depressions, unlike previous inbred populations which have experienced severe colony crash events (Rhode *et al.* 2020). The low-sequence diversity and strongly positive Tajima D statistic are consistent with a recent population bottleneck. Reduced nucleotide diversity and Tajima D on chromosome five are consistent with patterns of extended homozygosity, particularly within the 79.9 to 97.5 Mb region (Figure 4). An excess of rare alleles in this region may indicate a potential selective sweep. High proportions of short ROH indicates many recombination events in the life history of this population during long-term domestication (Gibson *et al.* 2006). However, long ROH indicates recent inbreeding, perhaps as a result of the founder effect during the establishment of the EVE line from an extremely small population (Ceballos *et al.* 2018). Nonetheless, it remains difficult to disentangle the effects of drift and selection in reducing genetic diversity on this chromosome.

Functional annotation of the 17.6 Mb region of interest containing 241 genes identified Gene Ontology (GO) terms for 105 (43.57%) genes involved in biological processes. Representation of genes with GO terms was disproportionally allocated categories under metabolism including organic substrate and nitrogen compound metabolism (Supplementary Figure S6). Ortholog analysis of this region using the EggNOG database provided annotations for 139 genes (57.68%) predominantly involved in the metabolism of amino acids, carbohydrates, secondary metabolites, and lipids (Supplementary Table S8). Interestingly, these genes include a cluster of cytochrome P450’s, perhaps involved in response to the metabolism of many highly toxic and diverse exogenous compounds enabling the generalist diet of BSF (Scott and Wen 2001; Schuler and Berenbaum 2013; Dobermann *et al.* 2019). Further candidates within this region include a pupation gene crucial in the onset of pupal casing development for metamorphosis and the well-studied *yellow* gene (Wittkopp *et al.* 2002). Integral to the structural formation of the cuticle, the *yellow* gene has also been linked to changes in the larval mouthparts (Brehme 1941). However, in the absence of phenotype data and genetically differentiated wild populations, identifying the genetic basis of domestication is challenging. Further analysis, using population genetic techniques will further reveal the genetic basis of domestication in the BSF, with chromosome five being of particular interest. Identification of genetic changes associated with domestication will enhance the potential for selective breeding programs and the improvement of life-history traits through artificial selection.

### Sex chromosome identification

Chromosome one to six exhibited the coverage of an autosome, while chromosome seven showed approximately half the autosomal coverage in males, as expected for an X chromosome (Supplementary Figure S7). Closely related Diptera species are likewise male-heterogametic, supporting an XY sex-determination system in BSF. Unlike *Drosophila melanogaster*, the dot-like (Muller F-element) chromosome of BSF is not a redundant sex chromosome and will be of interest for chromosome evolution studies (Vicoso and Bachtrog 2013). We identified 337 genes located on the sex chromosome and provided functional annotation using ortholog identity to the EggNOG database of 170 genes (50.45%). Genes located on the sex chromosome showed an overrepresentation of categories involved in cellular processes and signaling (Supplementary Table S9).

### Conclusion

We used a combination of PacBio, 10X Genomics linked-reads and Hi-C analysis to assemble the first chromosome-scale BSF genome. Final genome size of 1.01 Gb was produced using 10X and Hi-C scaffolding to obtain contig and scaffold N50 values of 16.01 Mb and 180.46 Mb, respectively. We annotated 16,478 protein-coding genes using the BRAKER2 pipeline. This chromosome-level assembly provides a significant increase (>100-fold contiguity) in reference quality compared with the existing reference genome. Genomic characterization identified a sex chromosome and potential candidate regions for further genomic investigations. We also identify the inbreeding coefficient of the established reference population, demonstrating a potential method for inbreeding monitoring in commercial mass rearing BSF facilities. Availability of this novel chromosomal Stratiomyidae reference assembly will be of interest for comparative chromosomal and genome evolution studies across the Diptera, as well as aiding further research to characterize the genetic architecture behind commercially valuable traits of the BSF. The genome will also be of benefit for developing biotechnology resources for genetic manipulation to improve the efficient exploitation of this farmed insect.
